# Resection of a pure androgen secreting adrenal adenoma in a postmenopausal woman: a case report

**DOI:** 10.1093/jscr/rjad693

**Published:** 2024-01-04

**Authors:** Ahmad Dalal, Abdallah Dwayat, Natalie Khamashta, Mo’men Alashwas, Tariq Asi

**Affiliations:** Faculty of Medicine, Main Campus, Al-Quds University, Abu Dis, Jerusalem, Palestine; Faculty of Medicine, Main Campus, Al-Quds University, Abu Dis, Jerusalem, Palestine; Faculty of Medicine, Main Campus, Al-Quds University, Abu Dis, Jerusalem, Palestine; Faculty of Medicine, Main Campus, Al-Quds University, Abu Dis, Jerusalem, Palestine; Department of Urology, Al-Quds University, Palestine Medical Complex, Jerusalem, Palestine

**Keywords:** pure androgen secreting adrenal tumor, PASAT, postmenopausal hyperandrogenism, adrenal adenoma, virilizing tumor

## Abstract

Pure androgen secreting adrenal tumors are exceedingly rare, presenting in higher numbers in women compared with men, and are particularly rare in women of postmenopausal age. Postmenopausal hyperandrogenism is usually ovarian or adrenal in origin, with tumors representing an uncommon cause, which are more frequently ovarian but could also be adrenal. Herein we present a case of a 61-year-old postmenopausal woman, who had suffered multiple reproductive disturbances, presenting with a 10-year history of virilizing symptoms, most bothersome of which was generalized hirsutism, alongside clitoromegaly, irritability, and voice deepening. Work-up of the patient revealed a 1.5 cm left adrenal mass, which was removed through laparoscopic total adrenalectomy. Postoperatively, the patient’s androgen levels dropped significantly. An adrenal androgen secreting tumor is a can't miss diagnosis that should always be considered in the evaluation of postmenopausal women with hyperandrogenism, alongside the more common etiologies. Regular hormonal follow-up is essential.

## Introduction

Pure androgen secreting adrenal tumors (PASATs) represent a rare uro-oncological entity. Epidemiology and natural behavior include being more common in females with a male-to-female ratio of 1:4. They have been reported in both children and adults and manifest as either benign or malignant. Cases of postmenopausal women represent a rarity among these exceedingly uncommon tumors [1–4].

## Case report

A 61-year-old female presented to the outpatient clinic complaining of abnormal facial and body hair growth for the last 10 years.

The thick and coarse hair growth involved her entire body, most noticeably over the face. These changes were accompanied with irritability and mood swings which according to her were in contrast to her laid-back personality. She also started experiencing fatigue and general weakness, in addition to swelling of the face and hands. She denied voice deepening but reported voice fatigability.

Regarding her reproductive history, the patient had regular periods starting at the age of 14; however, she had multiple disturbances throughout her adult life. Most notably, inability to conceive similar to all of her sisters, and paternal aunts. Menopause started early at the age of 40, followed by bothersome frequent hot flashes that lasted for ~14 years afterwards. Shortly after the onset of her menopause, she was diagnosed with polycystic ovarian syndrome (PCOS). Otherwise, her medical history was insignificant.

**Figure 1:**
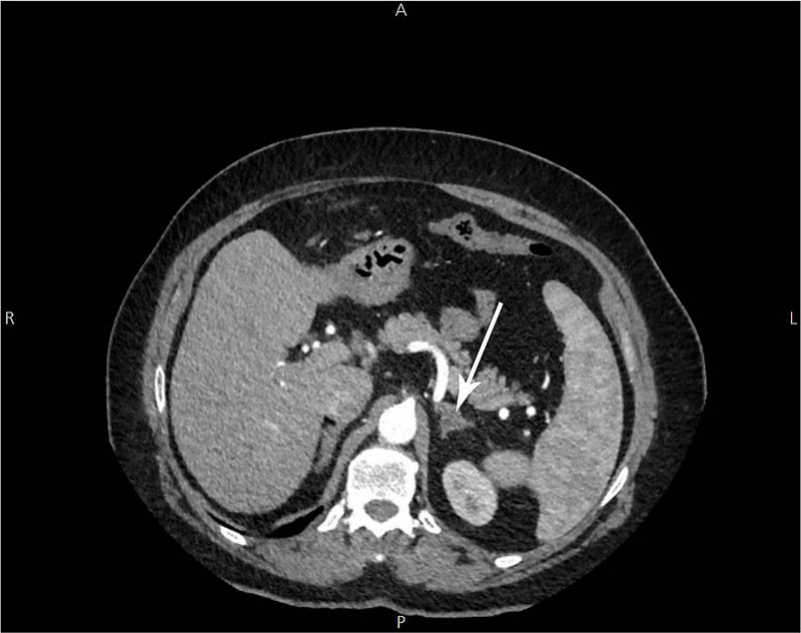
Arrow: a 1.5 cm non-enhancing hypodense nodule of the left suprarenal gland on CT imaging.

The patient consulted with a physician 2 years ago following failure of laser hair removal therapy done over 8 years. An abdominal and pelvic ultrasound was done which was unremarkable with normal intact ovaries. Contrast Computed Tomography (CT) of the abdomen and pelvis was performed (Fig. 1). It was significant for a non-enhancing hypodense nodule of the left suprarenal gland measuring 1.5 cm in diameter, which was mostly consistent with an adenoma. A 5.5 cm lesion, consistent with liver hemangioma, was seen at the lateral aspect of the right liver lobe.

Based on the clinical picture and imaging findings, hormone testing was done, and her testosterone level was found to be significantly elevated at 253.9 ng/dl (normal range for postmenopausal women, <7–48.9). Aldosterone, 24 hours urine free cortisol, plasma renin activity, follicle-stimulating hormone, and luteinizing hormone were all within the reference range.

Based on these findings, an androgen-secreting adrenal tumor was suspected. After which, she followed up with an endocrinologist for 6 months without improvement.

The patient was later referred to the urology clinic for further evaluation. On general examination, the patient appeared irritable and had a noticeably deep voice. Most remarkable exam findings included widely distributed hirsutism involving the entire body and clitoromegaly. Blood pressure was normal, and she had no palpable masses, acne, or cushingoid features. Otherwise, the exam was unremarkable.

Based on the patient's history, exam findings and previous investigations, that effectively excluded an exogenous or an ovarian etiology, the patient was scheduled for a laparoscopic left adrenalectomy for resection of the adrenal mass seen on imaging, as the suspected origin of hyperandrogenism.

The surgery went well with no complications, and the patient only complained of pain at the surgical site. She was discharged 2 days later in very well-condition. Testosterone levels 3 weeks postoperatively dropped to 9.6 ng/dl. Pathology reported a 1.5 cm adenoma in the left suprarenal gland. There was no sign of malignancy.

## Discussion

Adrenal tumors are present in 2% of the population. They are classified based on hormonal activity with <10% being active tumors. The majority of benign adrenocortical adenomas are inactive; however, active tumors usually secrete cortisol. Very rarely adrenal adenomas might purely secrete androgens or estrogens, but more commonly androgen hypersecretion is found along cortisol hypersecretion in adrenocortical carcinomas. Pure androgen-secreting adrenal tumors are considered rare in the population, and very rare in postmenopausal women with few cases reported [5].

In a French study conducted over 33 years (1970–2003), only 21 women (age 15–71) out of 801 patients who had adrenalectomies were found to have PASATs. Of those, only seven women were postmenopausal [3].

Virilizing symptoms occur in hyperandrogenic states. This includes acne eruptions, hirsutism, clitoromegaly, and voice deepening. Hirsutism is usually the main complaint among postmenopausal women. Hair is usually distributed over the face or trunk and male pattern hair loss might be present [6].

Androgen excess in postmenopausal women can be due to many etiologies, and can be classified based on origin, as ovarian and adrenal, or of tumor and non-tumor origin. The differential diagnosis includes PCOS, obesity-induced hyperandrogenic anovulation, ovarian hyperthecosis, androgen-secreting ovarian or adrenal tumor, cushing syndrome, congenital adrenal hyperplasia, and iatrogenic causes [6].

PCOS is one of the main causes of hyperandrogenism in women. Its pathophysiology involves a complex interplay of hormonal disturbances including insulin resistance and increased androgen levels. Our patient’s history of PCOS, familial female infertility, early menopause and her more recent diagnosis of an androgen secreting adenoma, might hint at a possible link between her conditions which might have co-existed for a period of time with an overlap of symptoms [6].

Surgical intervention, through total adrenalectomy involving removal of the adrenal gland as well as any present peri-adrenal tissue, is recommended for all cases of PASAT that present locally. As incomplete or delayed removal of malignant tumors infers a worse prognosis and significantly complicates management. If the etiology of the tumor is not certain, the standard approach remains total adrenalectomy, as partial adrenalectomy is contraindicated [2, 7].

PASATs are rare entities that are an unusual and often missed cause of postmenopausal hyperandrogenism. When evaluating patients with virilizing symptoms and elevated androgen levels, adrenal causes should always be in the back of a physician’s mind, alongside the more common etiologies. A history of multiple hormonal and reproductive disturbances poses a unique challenge for urologists and further complicates diagnosis. The treatment of choice for all cases of PASAT presenting locally remains total adrenalectomy. Regular hormonal follow up is pivotal to assess success of surgery and improvement of the patient’s condition.

## Conflict of interest statement

None declared.

## Funding

None declared.
